# FRED 2: an immunoinformatics framework for Python

**DOI:** 10.1093/bioinformatics/btw113

**Published:** 2016-02-26

**Authors:** Benjamin Schubert, Mathias Walzer, Hans-Philipp Brachvogel, András Szolek, Christopher Mohr, Oliver Kohlbacher

**Affiliations:** ^1^Center for Bioinformatics, University of Tübingen, Tübingen 72076, Germany; ^2^Department of Computer Science, Applied Bioinformatics, Tübingen 72076, Germany; ^3^Quantitative Biology Center, Tübingen 72076, Germany; ^4^Faculty of Medicine, University of Tübingen, Tübingen 72076, Germany; ^5^Max Planck Institute for Developmental Biology, Biomolecular Interactions, Tübingen 72076, Germany

## Abstract

**Summary:** Immunoinformatics approaches are widely used in a variety of applications from basic immunological to applied biomedical research. Complex data integration is inevitable in immunological research and usually requires comprehensive pipelines including multiple tools and data sources. Non-standard input and output formats of immunoinformatics tools make the development of such applications difficult. Here we present FRED 2, an open-source immunoinformatics framework offering easy and unified access to methods for epitope prediction and other immunoinformatics applications. FRED 2 is implemented in Python and designed to be extendable and flexible to allow rapid prototyping of complex applications.

**Availability and implementation:** FRED 2 is available at http://fred-2.github.io

**Contact:** schubert@informatik.uni-tuebingen.de

**Supplementary information:** Supplementary data are available at *Bioinformatics* online.

## 1 Introduction

The field of immunoinformatics has matured over the last decades. Epitope prediction methods are now widely used and have been successfully applied in many areas from basic immunological to translational research (Boisguérin *et al.*, 2014; Shukla *et al.*, 2015).

However, these applications often require complex pipelines combining multiple tools, multiple data sources and extensive pre- and post- processing. Furthermore, many of the HLA epitope prediction tools do not offer a unified interface and output format, which makes it difficult to use prediction methods interchangeably. One way to overcome these problems are web-based workbenches like the ones offered by IEDB (Vita *et al.*, 2015) or EpiToolKit (Schubert *et al.*, 2015). But often data volume, speed, or legal restrictions (e.g., concerning data privacy) prevent the use of such applications.

Also, web-based workbenches usually provide only limited or delayed integration of novel resources and methods. We therefore developed FRamework for Epitope Detection (FRED 2), an open-source, Python-based framework for computational immunology. FRED 2 is the (completely re-implemented) successor of FRED (Feldhahn *et al.*, 2009) and provides a unified interface to many prediction tools. We implemented routines covering data pre-processing, HLA typing, epitope prediction, epitope selection, as well as epitope assembly. FRED 2 is flexibly designed to allow easy extension. By building on top of popular modules such as BioPython (http://biopython.org) and Pandas (http://pandas.pydata.org), FRED 2 allows rapid prototyping of complex and innovative immunoinformatics applications.

## 2 Implementation

**Fig. 1:**
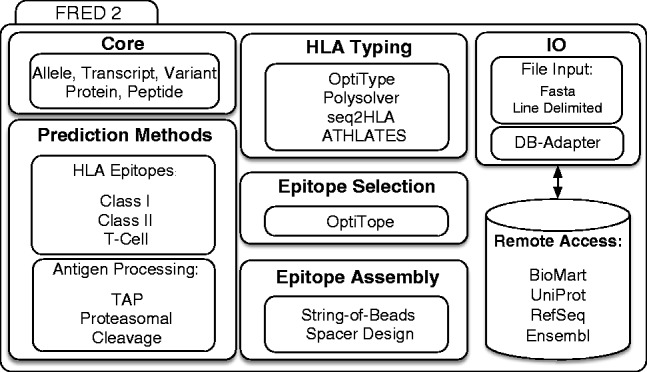
Schematic overview of FRED 2. FRED 2 is organized into modules dealing with epitope, cleavage and TAP prediction, HLA typing, epitope selection and assembly. The framework also offers accession to biological databases

FRED 2 covers four major areas of immunoinformatics: T-cell epitope prediction, epitope selection, epitope assembly and HLA typing (Fig. 1). Prediction methods are split into three packages *EpitopePrediction*, *TAPPrediction* and *CleavagePrediction*, each providing factory classes as entry points for the supported prediction methods. A detailed overview of the supported prediction methods can be found in Supplementary Table S1. OptiTope (Toussaint and Kohlbacher, 2009), a highly flexible mathematical framework capable of expression various aspects of epitope-based vaccines, was implemented for epitope selection. To enable epitope assembly, FRED 2 implements the traveling-salesperson (TSP) approach proposed by Toussaint *et al*. (Toussaint *et al.*, 2011) and OptiVac (Schubert and Kohlbacher, 2016) for string-of-beads design with optimal spacer sequences, which is similar to the approach taken in (Antonets and Bazhan, 2013). For HLA typing, FRED 2 provides wrapper methods for many HLA typing approaches, such as OptiType (Szolek *et al.*, 2014), Polysolver (Shukla *et al.*, 2015), seq2HLA (Boegel *et al.*, 2013) and ATHLATES (Liu *et al.*, 2013). FRED 2 also offers methods to interact with many biological databases like BioMart, UniProt, RefSeq and Ensembl. It provides support for handling sequence variations at all major biological levels, from transcript, protein, to peptide level. FRED 2 is open-source software and released under a three-clause BSD license. It was designed to be open and easily extendable by providing self explanatory interfaces so that implementation of new functionalities by a wider community can be easily accomplished.

## 3 Applications

A typical application of immunoinformatics is population-based vaccine design. In the following we show how to perform this task using FRED 2. Given a target population represented by their HLA alleles and virus proteins of interest, OptiTope (Toussaint and Kohlbacher, 2009) can be used to select the most immunogenic epitopes that are constrained to cover at least a fraction of HLA alleles and antigens. The immunogenicity of the epitopes can be approximated using NetMHC (Lundegaard *et al.*, 2008) or similar prediction tools supported by FRED 2. All prediction methods can interact with FRED 2s implementation of OptiTope, overcoming previous limitations of the tool (Toussaint and Kohlbacher, 2009). The described approach and the corresponding FRED 2 implementation can be found in Listing 1.

**Listing 1**: Population-based vaccine design with FRED 2

1. #read in virus proteins of interest

2. prots = IO.read_fasta(“./proteins.fasta”,in_type = Protein)

3. #read in HLA alleles of target population

4. hlas = IO.read_line(“./europe_hlas.txt”,in_type = Allele)

5. #generate 9mer peptides from proteins

6. peps = Generator.generate_peptides_from_proteins(prots,9)

7.

8. #predict binding affinity

9. netMHC = EpitopePredictionFactory(“NetMHC”)

10. aff = netMHC.predict(peps, alleles = hlas)

11. #initialize OptiTope and select up to 10 epitopes.

12. #assume a binding threshold of 500nM = 0.425 NetMHC score

13. opt = OptiTope(aff, threshold = {a:0.425 **for** a **in** hlas})

14. opt.set_k(10)

15. opt.activate_antigen_coverage_const(0.8)

16. opt.activate_allele_coverage_const(0.9)

17. selection = opt.solve()

Other tutorials in form of IPython notebooks, as well as detailed documentation of the source code can be found on FRED 2’s GitHub repository (http://fred-2.github.io).

## 4 Conclusion

We present FRED 2, a versatile immunoinformatics software framework enabling a unified interface to many tools, from epitope prediction, HLA typing, to epitope selection and assembly. Its openness and easy extensibility makes FRED 2 a perfect instrument for the development of advanced immunoinformatics pipelines that are needed for example in cancer immunotherapy development and other areas of personalized medicine.

## Funding

This project has received funding from the *European Union’s Horizon 2020 research and innovation programme* under grant agreement No. 633592 (APERIM). OK acknowledges funding from the Deutsche Forschungsgemeinschaft (SFB685/B1).

*Conflict of Interest*: none declared.

## Supplementary Material

Supplementary Data
