# Supplementary material for: FRED 2: an immunoinformatics framework for Python
Source: Bioinformatics. 2016 Feb 26;32(13):2044–6. doi: 10.1093/bioinformatics/btw113 (PMC4920123; doi:10.1093/bioinformatics/btw113)
Supplement: Supplementary Data [file supp_32_13_2044__index.html]

FRED 2: an immunoinformatics framework for Python — FRED 2: an immunoinformatics framework for Python — Supplementary Data 

# FRED 2: an immunoinformatics framework for Python

## Supplementary Data

files

- Supplementary Data - pdf file
