# Supplementary material for: FRED 2: an immunoinformatics framework for Python
Source: Bioinformatics. 2016 Feb 26;32(13):2044–6. doi: 10.1093/bioinformatics/btw113 (PMC4920123; doi:10.1093/bioinformatics/btw113)
Supplement: Supplementary Data [file supp_btw113_S1.pdf]

FRED 2 – An Immunoinformatics Framework for Python

Benjamin Schubert[1,2,*], Mathias Walzer[1,2], Hans-Philipp Brachvogel[1], András Szolek[1,2], Christopher Mohr[1,2],and Oliver Kohlbacher[1,2,3,4,5]

[1]Center for Bioinformatics, University of Tübingen, 72076 Tübingen, Germany

[2]Applied Bioinformatics, Dept. of Computer Science, 72076 Tübingen, Germany

[3]Quantitative Biology Center, 72076 Tübingen, Germany

[4]Faculty of Medicine, University of Tübingen, 72076 Tübingen, Germany

[5]Biomolecular Interactions, Max Planck Institute for Developmental Biology, 72076 Tübingen, Germany

Method	Version	Usage	Platform Compatibility	Reference
**MHC binding:**				
SYFPEITHI	1.0	T-cell epitope	Windows, Linux, Mac	(Rammensee, et al., 1999)
BIMAS	1.0	MHC-I binding	Windows, Linux, Mac	(Parker, et al., 1994)
SVMHC	1.0	MHC-I binding	Windows, Linux, Mac	(Dönnes and Elofsson, 2002)
ARB	1.0	MHC-I binding	Windows, Linux, Mac	(Bui, et al., 2005)
SMM	1.0	MHC-I binding	Windows, Linux, Mac	(Peters and Sette, 2005)
SMMPMBEC	1.0	MHC-I binding	Windows, Linux, Mac	(Kim, et al., 2009)
Epidemix	1.1	MHC-I binding	Windows, Linux, Mac	(Feldhahn, et al., 2009)
Comblib Sidney 2008	1.0	MHC-I binding	Windows, Linux, Mac	(Sidney, et al., 2008)
PickPocket*	1.1	MHC-I binding	Linux, Mac	(Zhang, et al., 2009)
NetMHC*	3.0, 3.4	MHC-I binding	Linux, Mac	(Lundegaard, et al., 2008)
NetMHCpan*	2.4, 2.8	MHC-I binding	Linux, Mac	(Hoof, et al., 2009)
HAMMER	1.0	MHC-II binding	Windows, Linux, Mac	(Sturniolo, et al., 1999)
TEPITOPEpan	1.0	MHC-II binding	Windows, Linux, Mac	(Zhang, et al., 2012)
NetMHCII*	2.2	MHC-II binding	Linux, Mac	(Nielsen, et al., 2007)
NetMHCIIpan*	3.0, 3.1	MHC-II binding	Linux, Mac	(Karosiene, et al., 2013)
UniTope	1.0	T-cell epitope	Windows, Linux, Mac	(Toussaint, et al., 2011)
NetCTLpan*	1.1	T-cell epitope	Linux, Mac	(Stranzl, et al., 2010)
**Cleavage Prediction:**				
ProteaSMM (C/S20)	1.0	Cleavage site	Windows, Linux, Mac	(Tenzer, et al., 2005)
PCM	1.0	Cleavage site	Windows, Linux, Mac	(Dönnes and Kohlbacher, 2005)
NetChop*	3.1	Cleavage site	Linux, Mac	(Nielsen, et al., 2005)
Ginodi	1.0	Cleavage fragment	Windows, Linux, Mac	(Ginodi, et al., 2008)
**TAP Prediction:**				
SVMTAP	1.0	TAP affinity	Windows, Linux, Mac	(Dönnes and Kohlbacher, 2005)
SMMTAP	1.0	TAP affinity	Windows, Linux, Mac	(Peters, et al., 2003)
Additive matrix method	1.0	TAP affinity	Windows, Linux, Mac	(Doytchinova, et al., 2004)
**Epitope Selection:**				
OptiTope[+]	1.0	Epitope selection for vaccine design	Windows, Linux, Mac	(Toussaint and Kohlbacher, 2009)
**Epitope Assembly:**				
TSP approach[+]	1.0	String-of-beads design	Windows, Linux, Mac	(Toussaint, et al., 2011)
Spacer design + TSP[+]	1.0	Spacer design	Windows, Linux, Mac	(Schubert and Kohlbacher, 2016)
**HLA Typing:**				
OptiType*	1.0	MHC-I typing	Linux, Mac	(Szolek, et al., 2014)

Polysolver*	1.0	MHC-I typing	Linux, Mac	(Shukla, et al., 2015)
Seq2HLA*	2.2	MHC-I/II typing	Linux, Mac	(Boegel, et al., 2013)
ATHLATES*	1.0	MHC-I/II typing	Linux, Mac	(Liu, et al., 2013)

* Installation of external software is required.

+ An integer linear programming solver such as CBC (https://projects.coin-or.org/Cbc) is required. For Epitope assembly the LKH approximation software (http://www.akira.ruc.dk/~keld/research/LKH/) is advised to use.

The integrated tools were chosen due to their wide spread use and high accuracy (Backert and Kohlbacher, 2015). Some methods mentioned by Backert et al. could not be integrated due to lack of publicly available stand-alone software or inadequate description of the method. However, FRED 2 was designed to be easily extendable by providing well defined interfaces as well as tutorials dedicated to developers (https://github.com/FRED-2/Fred2/wiki). We explicitly encourage users to contribute to FRED 2 and extend its capabilities by integrating new prediction methods or by extending its capabilities.

REFERENCES

Backert, L. and Kohlbacher, O. Immunoinformatics and epitope prediction in the age of genomic medicine. *Genome medicine* 2015;7(1):1-12.

Boegel, S., *et al.* HLA typing from RNA-Seq sequence reads. *Genome Medicine* 2013;4(12):102.

Bui, H.-H., *et al.* Automated generation and evaluation of specific MHC binding predictive tools: ARB matrix applications. *Immunogenetics* 2005;57(5):304-314.

Dönnes, P. and Elofsson, A. Prediction of MHC class I binding peptides, using SVMHC. *BMC bioinformatics* 2002;3(1):25.

Dönnes, P. and Kohlbacher, O. Integrated modeling of the major events in the MHC class I antigen processing pathway. *Protein science* 2005;14(8):2132-2140.

Doytchinova, I., Hemsley, S. and Flower, D.R. Transporter associated with antigen processing preselection of peptides binding to the MHC: a bioinformatic evaluation. *The Journal of Immunology* 2004;173(11):6813-6819.

Feldhahn, M., *et al.* FRED—a framework for T-cell epitope detection. *Bioinformatics* 2009;25(20):2758-2759.

Ginodi, I., *et al.* Precise score for the prediction of peptides cleaved by the proteasome. *Bioinformatics* 2008;24(4):477-483.

Hoof, I., *et al.* NetMHCpan, a method for MHC class I binding prediction beyond humans. *Immunogenetics* 2009;61(1):1-13.

Karosiene, E., *et al.* NetMHCIIpan-3.0, a common pan-specific MHC class II prediction method including all three human MHC class II isotypes, HLA-DR, HLA-DP and HLA-DQ. *Immunogenetics* 2013;65(10):711-724.

Kim, Y., *et al.* Derivation of an amino acid similarity matrix for peptide: MHC binding and its application as a Bayesian prior. *BMC bioinformatics* 2009;10(1):394.

Liu, C., *et al.* ATHLATES: accurate typing of human leukocyte antigen through exome sequencing. *Nucleic acids research* 2013;41(14):e142-e142.

Lundegaard, C., *et al.* NetMHC-3.0: accurate web accessible predictions of human, mouse and monkey MHC class I affinities for peptides of length 8–11. *Nucleic acids research* 2008;36(suppl 2):W509-W512.

Nielsen, M., Lundegaard, C. and Lund, O. Prediction of MHC class II binding affinity using SMM-align, a novel stabilization matrix alignment method. *BMC bioinformatics* 2007;8(1):238.

Nielsen, M., *et al.* The role of the proteasome in generating cytotoxic T-cell epitopes: insights obtained from improved predictions of proteasomal cleavage. *Immunogenetics* 2005;57(1-2):33-41.

Parker, K.C., Bednarek, M.A. and Coligan, J.E. Scheme for ranking potential HLA-A2 binding peptides based on independent binding of individual peptide side-chains. *The Journal of Immunology* 1994;152(1):163-175.

Peters, B., *et al.* Identifying MHC class I epitopes by predicting the TAP transport efficiency of epitope precursors. *The Journal of Immunology* 2003;171(4):1741-1749.

Peters, B. and Sette, A. Generating quantitative models describing the sequence specificity of biological processes with the stabilized matrix method. *BMC bioinformatics* 2005;6(1):132.

Rammensee, H.-G., *et al.* SYFPEITHI: database for MHC ligands and peptide motifs. *Immunogenetics* 1999;50(3-4):213-219.

Schubert, B. and Kohlbacher, O. Designing string-of-beads vaccines with optimal spacers. *Genome Medicine* 2016;8(1):1-10.

Shukla, S.A., *et al.* Comprehensive analysis of cancer-associated somatic mutations in class I HLA genes. *Nat Biotech* 2015;advance online publication.

Sidney, J., *et al.* Quantitative peptide binding motifs for 19 human and mouse MHC class I molecules derived using positional scanning combinatorial peptide libraries. *Immunome Res* 2008;4(2):7580-7584.

Stranzl, T., *et al.* NetCTLpan: pan-specific MHC class I pathway epitope predictions. *Immunogenetics* 2010;62(6):357-368.

Sturniolo, T., *et al.* Generation of tissue-specific and promiscuous HLA ligand databases using DNA microarrays and virtual HLA class II matrices. *Nature biotechnology* 1999;17(6):555-561.

Szolek, A., *et al.* OptiType: precision HLA typing from next-generation sequencing data. *Bioinformatics* 2014;30(23):3310-3316.

Tenzer, S., *et al.* Modeling the MHC class I pathway by combining predictions of proteasomal cleavage, TAP transport and MHC class I binding. *Cellular and Molecular Life Sciences CMLS* 2005;62(9):1025-1037.

Toussaint, N.C., *et al.* T-cell epitope prediction based on self-tolerance. In, *Proceedings of the 2nd ACM Conference on Bioinformatics, Computational Biology and Biomedicine*. ACM; 2011. p. 584-588.

Toussaint, N.C. and Kohlbacher, O. OptiTope—a web server for the selection of an optimal set of peptides for epitope-based vaccines. *Nucleic acids research* 2009;37(suppl 2):W617-W622.

Toussaint, N.C., *et al.* Universal peptide vaccines–Optimal peptide vaccine design based on viral sequence conservation. *Vaccine* 2011;29(47):8745-8753.

Zhang, H., Lund, O. and Nielsen, M. The PickPocket method for predicting binding specificities for receptors based on receptor pocket similarities: application to MHC-peptide binding. *Bioinformatics* 2009;25(10):1293-1299.

Zhang, L., *et al.* TEPITOPEpan: extending TEPITOPE for peptide binding prediction covering over 700 HLA-DR molecules. 2012.
